# Structure of the RecQ C-terminal Domain of Human Bloom Syndrome Protein

**DOI:** 10.1038/srep03294

**Published:** 2013-11-21

**Authors:** Sun-Yong Kim, Toshio Hakoshima, Ken Kitano

**Affiliations:** 1 Structural Biology Laboratory, Graduate School of Biological Sciences, Nara Institute of Science and Technology, 8916-5 Takayama, Ikoma, Nara 630-0192, Japan

## Abstract

Bloom syndrome is a rare genetic disorder characterized by genomic instability and cancer predisposition. The disease is caused by mutations of the Bloom syndrome protein (BLM). Here we report the crystal structure of a RecQ C-terminal (RQC) domain from human BLM. The structure reveals three novel features of BLM RQC which distinguish it from the previous structures of the Werner syndrome protein (WRN) and RECQ1. First, BLM RQC lacks an aromatic residue at the tip of the β-wing, a key element of the RecQ-family helicases used for DNA-strand separation. Second, a BLM-specific insertion between the N-terminal helices exhibits a looping-out structure that extends at right angles to the β-wing. Deletion mutagenesis of this insertion interfered with binding to Holliday junction. Third, the C-terminal region of BLM RQC adopts an extended structure running along the domain surface, which may facilitate the spatial positioning of an HRDC domain in the full-length protein.

RecQ helicases, a family of DNA unwinding enzymes that are conserved from bacteria to mammals, play a key role in protecting the genome against deleterious changes (for recent reviews^1^,^2^,^3^,^4^). Whereas the genomes of bacteria typically encode a single recQ gene, the human genome contains five recQ genes that encode RECQ1, Bloom syndrome protein (BLM), Werner syndrome protein (WRN), RECQ4 and RECQ5. Mutations in BLM and WRN are associated with the rare genetic diseases Bloom and Werner syndromes, respectively. Although the two diseases are commonly characterized by pronounced genomic instability and cancer predisposition, the clinical symptoms of Bloom and Werner syndromes are distinct: Bloom patients display severe growth retardation with short stature, immunodeficiency, sunlight sensitivity, and a predisposition to a wide spectrum of cancers, while Werner patients display features of accelerated aging^2^,^4^. The different clinical features of the disorders, and the fact that the functional loss of BLM or WRN cannot be compensated for by the presence of the other protein (or of other RecQ members), support the notion that BLM and WRN have distinct functions in cells.

A domain diagram of RecQ helicases is shown in Figure 1a. The largest and most highly conserved component of the RecQ family is the ATPase domain, which acts as an ATP-dependent DNA translocation module^5^. In addition, BLM and WRN share two conserved regions at the C terminus, referred to as the RecQ C-terminal (RQC) and helicase-and-ribonuclease D-C-terminal (HRDC) domains. Whereas the function of the HRDC domain remains unclear, the recently determined co-crystal structure of a WRN RQC-DNA complex revealed a central role for the RQC winged-helix motif in recognizing, binding, and unwinding DNA at branch points^6^.

However, the RQC domain is one of the most divergent components of RecQ family proteins, and the sequence identity between BLM RQC and WRN RQC is only ~10%. It is likely that the variability in RQC sequences yields structural and functional differences between BLM and WRN and hence contributes to the onset of Bloom and Werner syndromes. In this study, we have determined the first three-dimensional structure of the BLM RQC domain. The structure and our subsequent biochemical analyses of the domain suggest a mechanism whereby the BLM RQC domain (and the BLM HRDC domain) catalyzes branch migration of a Holliday junction (HJ).

## Results

### Purification and structural determination

**Figure 1:**
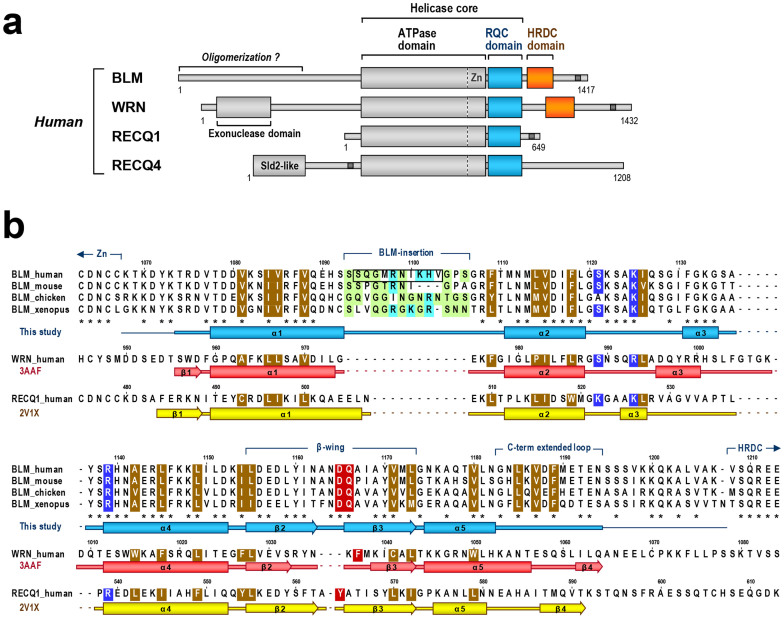
Amino acid sequences of RQC domains in the RecQ family. (a) Domain diagram of human RecQ helicases. The RQC and HRDC domains are colored blue and orange, respectively. The zinc-binding subdomain (Zn), which is unique to the RecQ family of proteins and is often combined with the RQC sequence, is depicted in the C terminus of the ATPase domain, based on the latest structural data^1^,^9^,^31^,^38^. The ATPase and RQC domains generally comprise a “helicase core” of the RecQ family^1^,^6^,^39^. Nuclear localization signals are depicted as dark gray bars. The N-terminal region of BLM may be involved in oligomerization^40^, whereas WRN possesses an exonuclease domain at the N terminus. The fifth human RecQ member, RECQ5, which is expressed as three different splice variants^1^, is omitted here. (b) Sequence alignment of RQCs of BLM (from four animal species), WRN, and RECQ1. The RQC sequences are found only in the RecQ family of proteins^3^. The multiple alignment was retrieved from the Pfam (family ID: 09382) and NCBI databases and manually modified based on the three-dimensional structures; the secondary-structure elements of BLM (colored blue; present work), WRN (red; PDB ID of 3AAF)^6^, and RECQ1 (yellow; 2V1X)^9^ are shown below each sequence. In BLM, disordered regions at the N and C termini are shown as thin lines, and identical residues among the species are denoted by asterisks. The small and/or polar residues of the BLM-insertion are light green, and the basic residues are light blue. The aromatic residues capping the β-wings of WRN (Phe1037) and RECQ1 (Tyr564), and the corresponding residues of BLM (Asp1165 and Gln1166) are red (Figure 3d). The conserved hydrophobic residues that comprise the RQC folding core are brown. In BLM, the amino acids that were subjected to deletion mutagenesis (loopΔ10) are boxed, while the three DNA-binding residues Ser1121, Lys1125, and Arg1139 are blue. Gly1120, which is strictly conserved among the members, is important to provide the α2–α3 loop with the flexibility required for DNA interaction^6^,^41^.

Recently, our group^7^ and another group^8^ independently determined the first three-dimensional structure of a BLM fragment, an NMR structure of the human BLM HRDC domain. The structure revealed that the HRDC fold of BLM starts at a.a. 1208, thus limiting the C terminus of the RQC domain to a more N-terminal amino acid. On the other hand, the crystal structure of the RECQ1 helicase core showed that a cysteine cluster ^475^CDNC^478^ prior to the RQC winged-helix participates in the zinc-binding subdomain, a distinct structural fold that is tightly packed against the ATPase domain^9^. These amino acids are conserved in BLM as ^1063^CDNC^1066^ (Figure 1b), suggesting that the RQC winged-helix of BLM starts at around a.a. 1070. Based on these observations, we generated an expression construct encoding a.a. 1068–1209 of human BLM, which covers the entire sequence of the RQC winged-helix domain. The fragment was overexpressed in *E. coli* and purified to homogeneity without any degradation (Supplementary Fig. S1). Crystals were obtained in conditions that included either a phosphate ion or an arsenate ion (Supplementary Fig. S2). The two crystals, which belong to the same space group, were employed for X-ray data collection. Phases were determined using the multiwavelength anomalous dispersion (MAD) method with a selenomethionine (SeMet) crystal.

### Overall structure of BLM RQC

**Figure 2:**
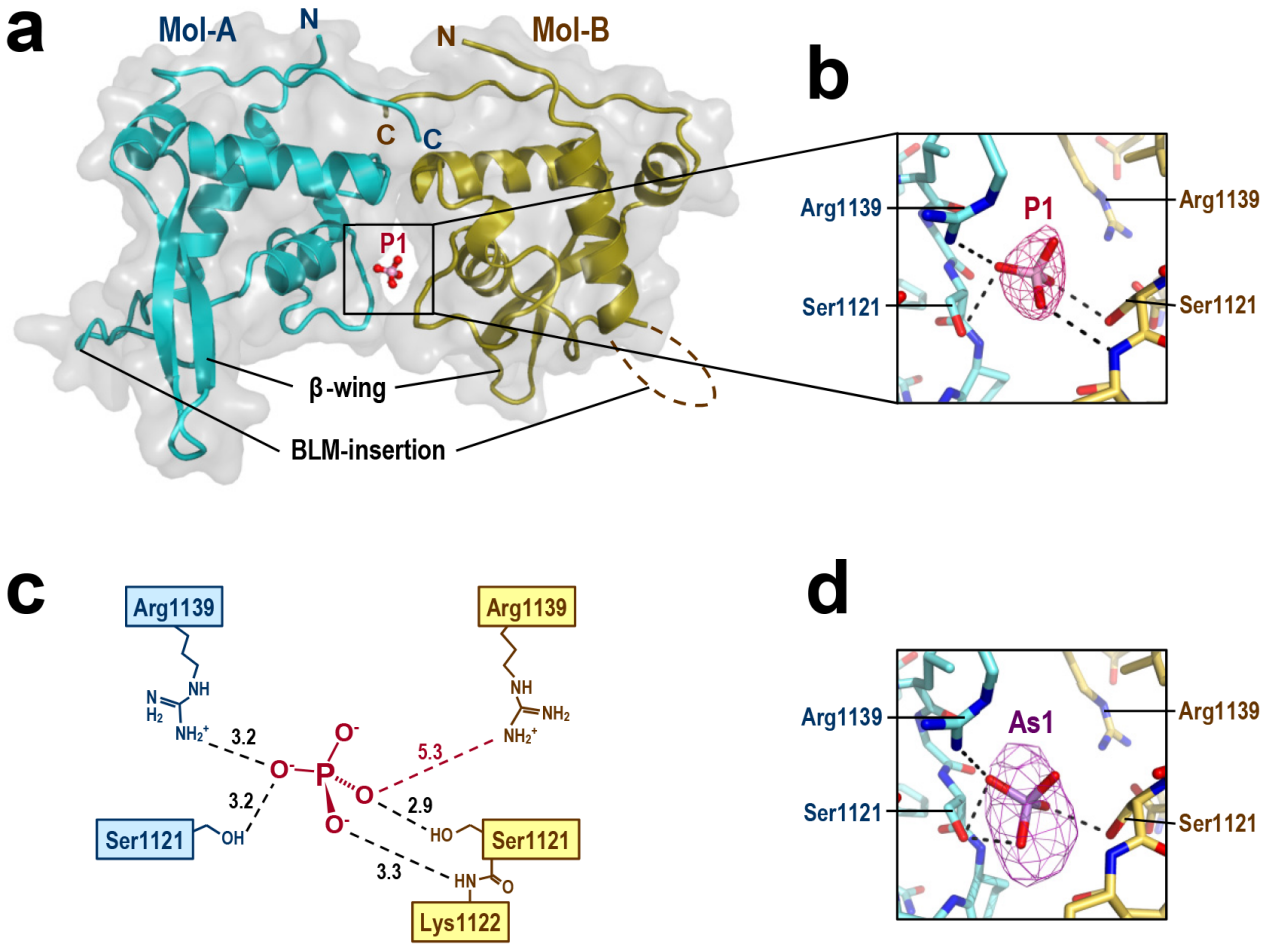
Structure of human BLM RQC domain bound to phosphate ion. (a) Crystal structure of the dimerized BLM RQC domains (Mol-A and -B, in blue and yellow, respectively) complexed with a phosphate ion (P1, in a stick model). The molecular surfaces of each domain are shown in transparent gray. (b) Close-up view of the interactions between P1 and the two RQC domains. Intermolecular hydrogen bonds within a distance of 3.3 Å are shown as dashed lines. Electron density (omit *F*o-*F*c map contoured at 5.0 σ) for P1 is superimposed. (c) Schematic representation of (b). Hydrogen bond lengths (Å) are labeled in black. Arg1139 of Mol-B is further from P1 (5.3 Å; red dashed line). (d) Close-up view of the arsenate ion (As1) in the BLM RQC-arsenate complex. An anomalous difference Fourier map contoured at 5.0 σ is superimposed.

The structures of human BLM RQC complexed with a phosphate ion (Figure 2a–c) and an arsenate ion (Figure 2d) were determined at resolutions of 2.7 and 2.9 Å, respectively (Table 1). The asymmetric unit of each crystal contains two RQC monomers (Mol-A and -B), which are bridged to each other by the ion (P1 or As1) shared at the interface. These structures demonstrate that P1 and As1 play a key role in lattice formation. Consistently, the phosphate or arsenate ion was a critical determinant of crystallization, and could not be replaced by other salts. For the arsenate ion complex, the X-ray data were also collected at the peak wavelength of arsenate (1.0439 Å) to calculate the anomalous difference Fourier map. The map gave a strong density surrounding the ion (Figure 2d), confirming the presence of the arsenate ion in this position.

**Figure 3:**
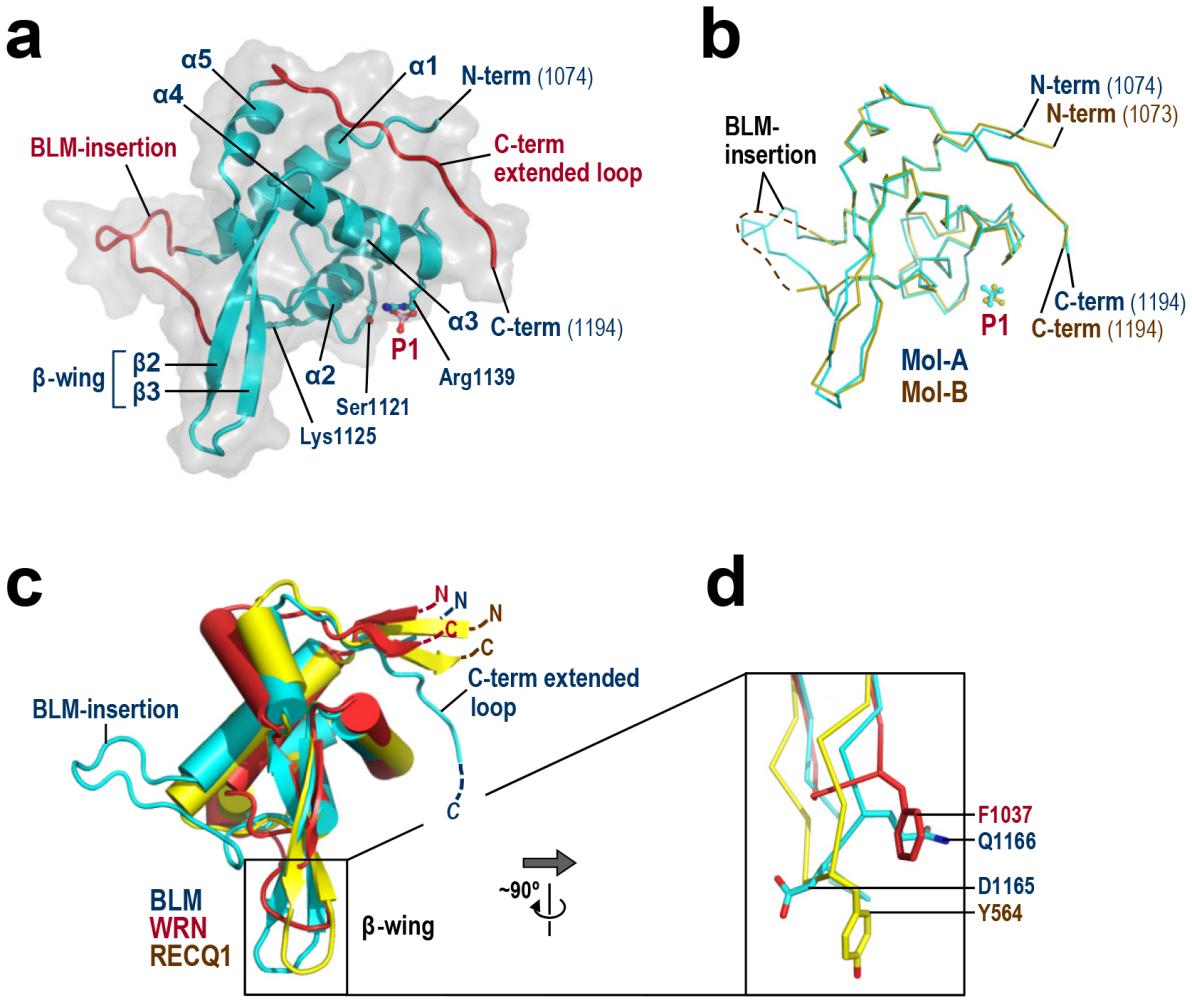
Monomeric structure of BLM RQC. (a) Structure of Mol-A. Secondary-structure elements (five α-helices and two β-strands) are labeled. The BLM-insertion (a.a. 1093–1106) and C-term extended loop (a.a. 1183–1194) are colored red. P1 and the three DNA-binding residues Ser1121, Lys1125 and Arg1139 (subjected to point mutations in Figure 7) are shown as stick models. (b) Superimposition of Mol-A (blue) and -B (yellow). The BLM-insertion of Mol-B (dashed line) is disordered due to the lack of lattice contact. (c) Structural comparison of the RQC domains of BLM (present structure, in blue), WRN^6^ (red) and RECQ1^9^ (yellow). The N and C termini of each molecule are labeled. The BLM RQC domain comprises 121 amino acids, and is slightly larger than those of WRN (109 a.a.) and RECQ1 (108 a.a.). (d) Close-up view of the β-wings of BLM, WRN and RECQ1 (after ~90° rotation along the y-axis). BLM residues Asp1165 and Gln1166 spatially overlap with RECQ1 Tyr564 and WRN Phe1037, respectively.

Figure 3a shows the monomeric structure of Mol-A bound to the phosphate ion. The 121 residues (a.a. 1074–1194) of human BLM fold into a winged-helix motif, a subset of the helix-turn-helix superfamily^10^. The structure of the second molecule (Mol-B), which is illustrated in Figure 3b, is essentially the same except that the looping-out region (referred to as the BLM-insertion) is disordered.

### β-wing

Figure 3c shows a structural superimposition of the BLM RQC domain with those of WRN^6^ and RECQ1^9^. The previously determined structure of a WRN RQC-DNA complex revealed that the RQC domain binds duplex DNA in an unconventional way relative to other characterized winged-helix and other helix-turn-helix proteins, and thereby splits the double-stranded DNA from the duplex terminus^6^. In particular, the prominent hairpin structure β2–β3, which corresponds to a so-called β-wing of the winged-helix fold^10^, directly catalyzes strand separation: the Phe1037 side-chain at the tip of the WRN β-wing (Figure 3d) wedges between the last paired and first unpaired bases, resulting in a loss of base-base stacking and separation of both strands^6^. The β-wing of RECQ1 is also capped by an aromatic residue, Tyr564, and mutagenesis studies of RECQ1^9^,^11^ and WRN^12^ have confirmed the importance of each aromatic residue in the helicase catalytic reactions.

In contrast, the β-wing of BLM is capped by acidic (Asp1165) and polar (Gln1166) residues (the ^1165^DQ^1166^ motif). These two residues constitute the structural counterparts of RECQ1 Tyr564 and WRN Phe1037, respectively. This analysis suggests that the strand-separation mechanism of BLM is somewhat different from those of WRN and RECQ1. An electron density map of the BLM β-wing is shown in Supplementary Fig. S3.

### BLM RQC is a monomer and binds to DNA in solution

**Figure 4:**
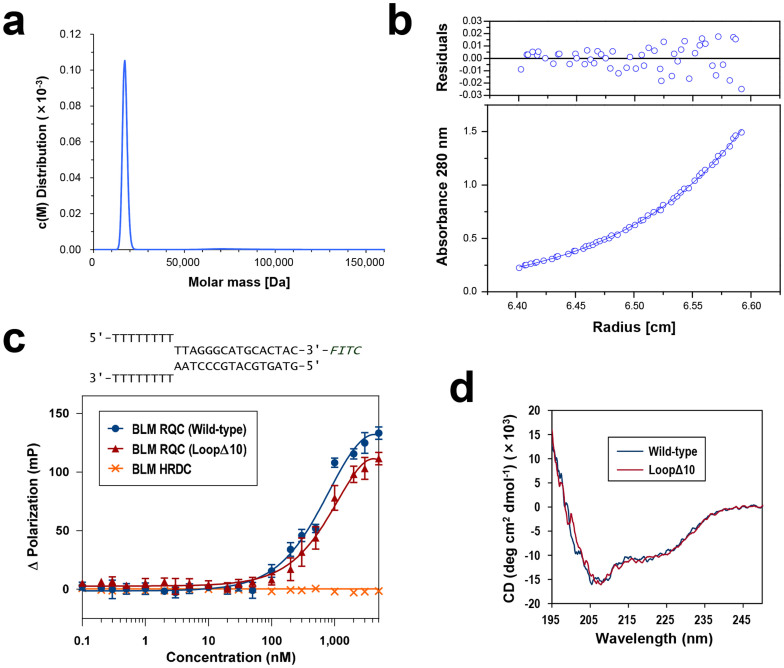
Ultracentrifugation, DNA-binding assay, and CD spectra of BLM RQC. (a) Ultracentrifuge sedimentation-velocity analysis of BLM RQC. The molar mass distribution curve obtained from the experiment shows a single peak at 17.2 kDa. (b) Ultracentrifuge sedimentation-equilibrium analysis of BLM RQC. Observed absorbance at 67 μM protein concentration is plotted against the radial distance. The best-fit curve from a single-species model is overlaid, yielding a molecular mass of 14.4 kDa. The upper part shows the residuals expressed as the difference between the experimental and the fitted data. Comparable results were obtained for 34 and 200 μM protein concentrations. (c) DNA-binding assay examined by fluorescence polarization. The forked duplex sequence (FITC-labeled) used in the assay is shown at the top. Interactions between the DNA and BLM RQCs in GST-free form (wild-type and loopΔ10 mutant; blue circles and red triangles, respectively) were examined with increasing protein concentrations from 0 to 5 μM. The BLM HRDC domain (a.a. 1200–1295; orange crosses)^7^ was also purified and used as a negative control in the experiment. (d) CD spectra of BLM RQC wild-type and the loopΔ10 mutant. Both samples yielded almost identical plots.

The oligomeric state of BLM is currently a matter of debate. In the absence of DNA, the full-length protein forms a hexameric and/or tetrameric ring structure^13^; however, the oligomer appears to dissociate when BLM acts on a DNA substrate^14^. To determine the precise assembly state of BLM RQC in solution, we employed analytical ultracentrifugation. First, sedimentation-velocity analysis yielded a monodisperse peak around 17.2 kDa in an estimation of molar mass distribution (Figure 4a), suggesting that the domain, with a calculated mass of 16.3 kDa, is present exclusively as a monomer. Second, sedimentation-equilibrium analysis gave a molecular mass of 14.4 kDa (Figure 4b), which is also concordant with the mass of the monomer. In summary, the isolated BLM RQC domain exists as a monomer in solution, as reported for the WRN RQC domain^6^,^15^.

We next examined the DNA-binding ability of BLM RQC in a fluorescence polarization assay. This experiment used a forked duplex, which is known to be a substrate of full-length BLM^1^,^3^. As shown in Figure 4c, BLM RQC (blue circles) binds to DNA in the nanomolar range, with an estimated dissociation constant of around 700 nM. The weaker interaction than that displayed by WRN RQC, which has a dissociation constant of 108 nM with the same forked duplex^6^, is compatible with a recent study reporting moderate interactions between full-length BLM and diverse DNA structures^16^.

### Phosphate ion position suggests the DNA-binding mode of BLM RQC

Since the BLM RQC domain binds to DNA in solution, we predicted that the phosphate ion P1 in the crystal structure might mimic one of the phosphate groups in the DNA substrate. As schematically represented in Figure 2c, P1 is bound by Mol-A and -B through nearly identical interactions, even though the two molecules are crystallographically independent of each other. The side chains of Ser1121 in both Mol-A and -B form hydrogen bonds with P1, while Arg1139 only in Mol-A forms a salt bridge with the ion; in Mol-B, Arg1139 is less close to P1 (5.3 Å), and instead a main-chain nitrogen of Lys1122 ligates the ion. The somewhat distorted binding symmetry between Mol-A and -B is due to the limited space within the interface, which only allows binding of a single phosphate ion.

**Figure 5:**
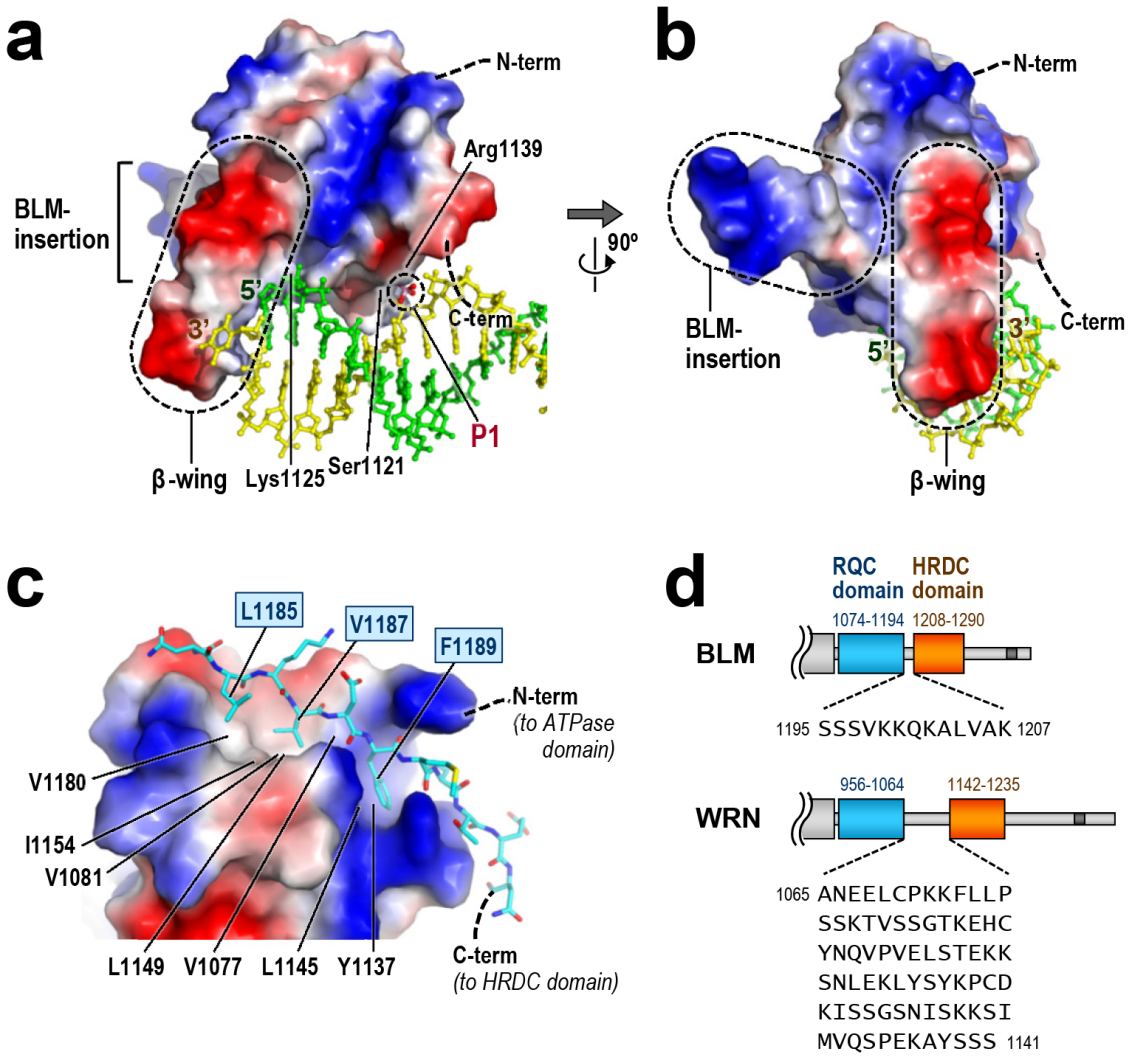
β-wing, BLM-insertion, and C-term extended loop. (a) Binding model of BLM RQC to a simple DNA duplex. Electrostatic surface potential of BLM RQC, colored for positive (blue), negative (red) and neutral (white) charges, is shown in an orientation similar to that in Figure 3a, while the structure of the DNA duplex from the WRN RQC-DNA complex^6^ is superimposed in green (5′-strand) and yellow (3′-strand) using the RQC domains as a reference (Figure 3c). The β-wing and phosphate ion P1 are encircled by dashed lines. (b) A view after 90° rotation along the y-axis (BLM RQC is in an orientation similar to that in Figure 3c). The BLM-insertion (additionally encircled by a dashed line) extends at ~90° to both the β-wing and the DNA duplex. (c) C-term extended loop (a.a. 1183–1194, in a stick model) packed against the RQC core (shown as an electrostatic surface potential). Orientation of the molecule is similar to that in Figure 3a. The three nonpolar side-chains on the C-term extended loop, Leu1185, Val1187 and Phe1189, are tightly packed against the hydrophobic groove of the RQC core (formed by Val1077, Val1081, Tyr1137, Leu1145, Leu1149, Ile1154 and Val1180). The interaction also includes seven hydrogen bonds (Supplementary Fig. S4). (d) Comparison of the RQC-HRDC linker regions in BLM and WRN. The domain boundaries were determined from the three-dimensional structures of BLM RQC (present structure), BLM HRDC^7^,^8^, WRN RQC^6^,^15^ and WRN HRDC^17^.

In Figure 5a and b, we have superimposed a duplex structure from the WRN RQC-DNA complex^6^ onto the surface potential of BLM RQC. In this binding model, BLM RQC interacts with the duplex terminus in a sequence-independent manner. Interestingly, the position of P1 strikingly overlaps with one of the backbone phosphates of the 3′-strand DNA (yellow). In the previous structure, this same phosphate group was bound by WRN RQC through similar interactions: the side chain of WRN Ser989 (corresponding to BLM Ser1121; Figure 1b) and the main-chain nitrogen of the next residue (corresponding to BLM Lys1122) ligate this phosphate group^6^. Therefore, the P1 position is compatible with the present binding model of BLM RQC. In addition, Lys1125, another conserved amino acid of BLM, is adjacent to a backbone phosphate of the 5′-strand DNA (green in Figure 5a). This is also compatible with the established functional importance of WRN Arg993 (the counterpart of BLM Lys1125), which plays a major role in both DNA-binding^6^ and unwinding^12^.

### BLM-insertion

The present structure showed that BLM RQC includes a 14-residue BLM-specific insertion, ^1093^SSQGMRNIKHVGPS^1106^, between the N-terminal helices α1 and α2 (BLM-insertion; Figure 3a). This region forms a looping-out structure, which protrudes from the domain surface at ~90° to the β-wing. In the aligned sequences (Figure 1b), the BLM-insertion is common to all BLMs from the animal species examined, although its sequences and numbers of amino acids vary (human and chicken insertions comprise 14 residues, while those of mouse and *Xenopus* include 11 and 13 residues, respectively). It is noteworthy that they are all rich in the small and/or polar residues (Gly, Ser, Thr, Asn and Gln; colored in light green) as well as the basic residues (Arg, Lys and His; light blue). Importantly, no acidic residue occurs in this region. As a consequence, the surface potential of the BLM-insertion is electropositive (Figure 5b), which is in sharp contrast to the electronegativity of the β-wing.

The present binding model (Figure 5b) suggests that the BLM-insertion is not involved in the interaction with duplex DNA. To test this, we purified a deletion mutant of BLM RQC in which ten of the BLM-insertion residues were removed (loopΔ10; the deletion mutagenesis target is boxed in Figure 1b) and examined its interaction with the forked duplex. As depicted in Figure 4c, loopΔ10 (red triangles) exhibited a similar binding profile to that of the wild-type domain, a result that is also consistent with the present binding model of BLM RQC. The circular dichroism (CD) spectrum analysis of the protein samples (Figure 4d) indicates that the loopΔ10 mutant remains structured in solution.

### C-term extended loop

BLM RQC is also distinct from other RQC domains in lacking the secondary structures β1 and β4, which are common to the N and C termini of previously determined structures (Figures 1b and 3c). Instead, the C-terminal region of BLM RQC adopts a unique extended structure, which traverses the domain surface (C-term extended loop). The region forms no particular secondary structure, but the extended conformation is stabilized by extensive interactions with the domain core. In particular, the hydrophobic side-chains of Leu1185, Val1187 and Phe1189 on the C-term extended loop are buried deep in the hydrophobic groove of the domain core (Figure 5c). These three residues are highly conserved (Figure 1b), suggesting that the extended structure is common to all BLMs.

In the full-length protein, the C-term extended loop is followed by the 13 amino acids ^1195^SSSVKKQKALVAK^1207^, which tether the C-terminal HRDC domain (Figure 5d). Interestingly, this linker is considerably shorter than that of WRN (77 residues). In contrast to WRN, whose HRDC domain is spatially well separated from the helicase core^17^, the BLM HRDC domain appears to be much closer to its RQC domain.

### Binding model to HJ

BLM acts preferentially on recombination and repair intermediates containing HJs to promote efficient branch migration^18^. In particular, BLM acts in concert with topoisomerase IIIα to resolve double HJs without the formation of crossover products^19^,^20^,^21^. This process, referred to as double HJ dissolution, is crucial for suppressing the sister-chromatid exchanges that cause early neoplastic transformation of cells^3^,^4^.

**Figure 6:**
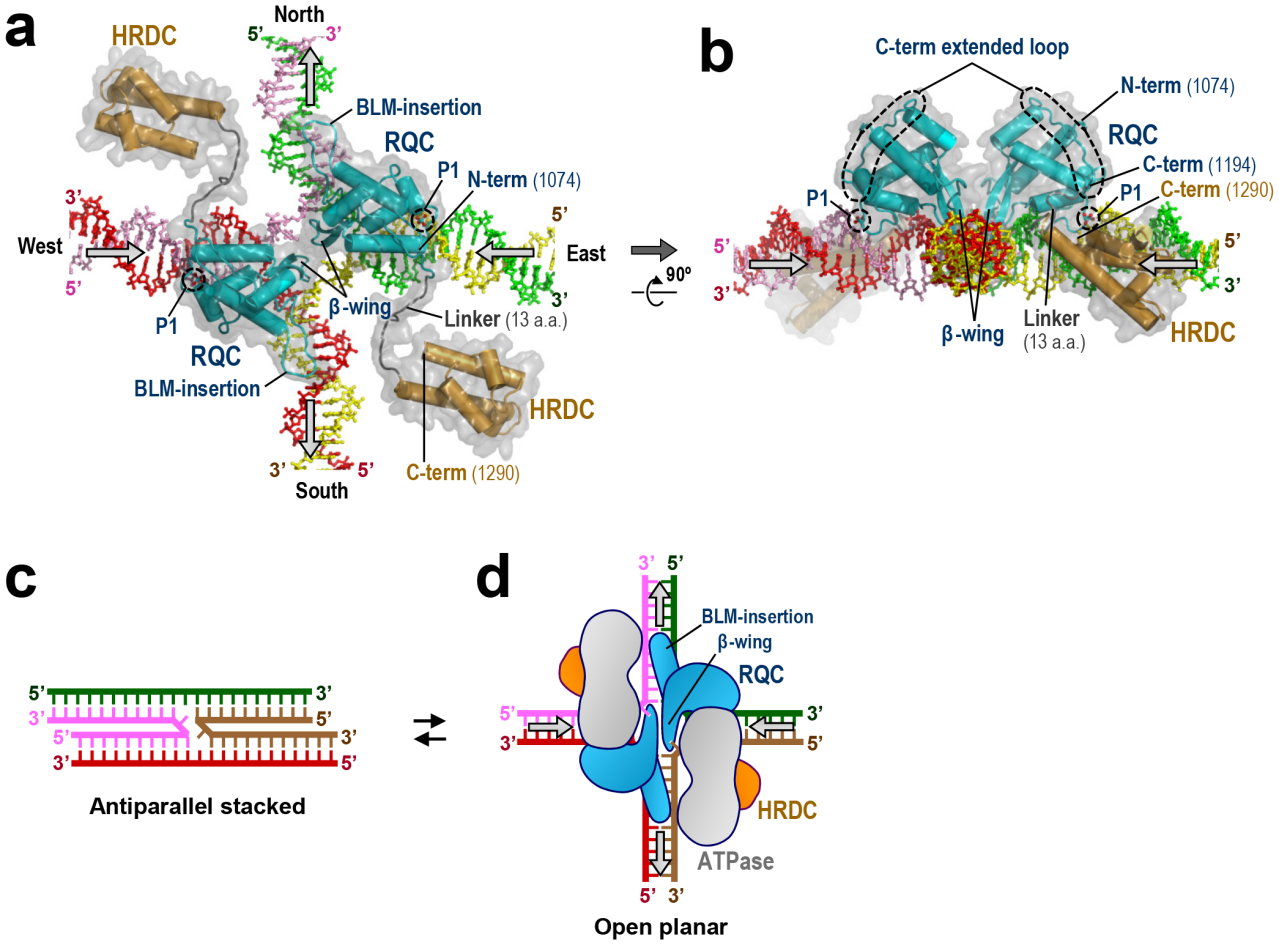
Binding model of BLM RQCs to HJ. (a) Structural model of BLM RQCs (blue) bound to HJ. The BLM HRDC domains (orange)^7^ are connected to each RQC C terminus with the 13-a.a. linker (gray). The positions of P1 overlap with one of the phosphate groups in each of the east and west arms (yellow and pink strands, respectively). The arrows represent the directions of DNA translocations required for branch migration. (b) View after 90° rotation along the x-axis. The C-term extended loops (encircled by dashed lines) run along the surface of each RQC domain, and the C termini (a.a. 1194) of the RQCs are adjacent to the east-west arms of HJ. (c) Schematic representation of HJ in the anti-parallel stacked conformation. (d) Schematic representation of (a), with HJ in the open planar conformation. Putative configurations of the BLM ATPase domains are also depicted in gray.

In Figure 6a and b, we present a binding model of BLM RQC to a HJ, which is based on a previous docking simulation of WRN RQC bound to a HJ^6^. In this model, two BLM RQC molecules bind the east and west arms of the HJ, while the BLM-insertion from each molecule runs in parallel with the north (green and pink) or south (red and yellow) arm. Since the BLM-insertion is electropositive, ionic interactions probably occur between the BLM-insertion and backbone phosphates of the HJ.

Meanwhile, the acidic β-wings from the two molecules fit well into the central hole of the HJ, displaying no steric hindrance with each other or DNA. This is reminiscent of the acidic hairpin of bacterial RuvA, a protein that also catalyzes branch migration of HJs (reviewed in^22^). By analogy with the proposed mechanism for RuvA, negative charges on the BLM β-wings may serve to repel the DNA backbones from the junction center (and/or to repel the other β-wing within the same hole) by electrostatic repulsion. The resultant mechanical force to enlarge the hole could result in the disruption of base pairs near the crossover point, thereby enhancing strand-exchange reactions.

### Mutagenesis binding assays with HJ

**Figure 7:**
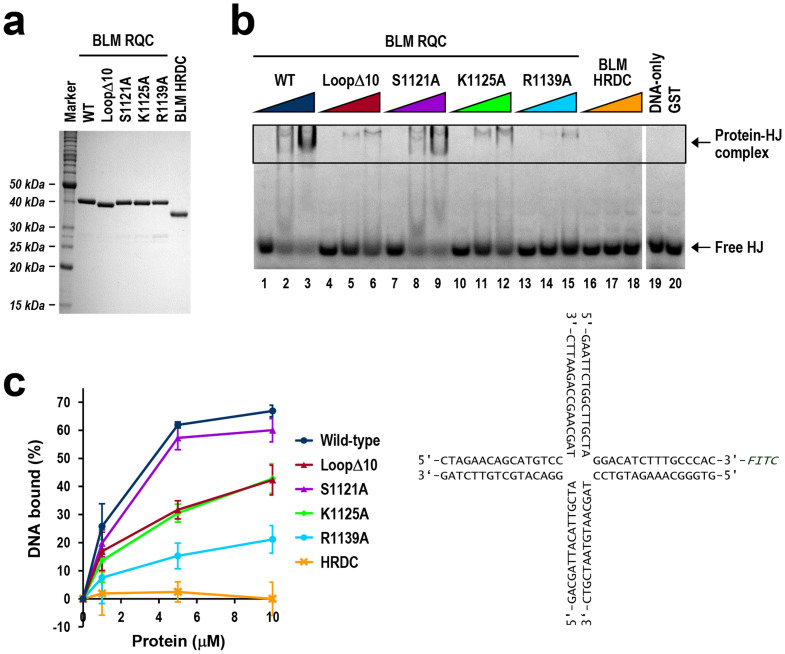
Mutagenesis binding assays of BLM RQC with HJ. (a) SDS-PAGE of BLM RQCs (wild-type, loopΔ10, S1121A, K1125A, and R1139A) and BLM HRDC in GST-fused form. One microgram of each protein was electrophoresed using a 13.8% polyacrylamide gel, which was stained with Coomassie Blue. (b) Mutagenesis binding assays with HJ by EMSA. BLM RQC wild-type in GST-fused form (lanes 1–3: 1, 5, and 10 μM, respectively), and four different variants (lanes 4–15) and BLM HRDC (lanes 16–18) with identical protein amounts, were each incubated with 0.2 μM FITC-labeled HJ (DNA sequences are shown below the gel). Free and protein-bound DNA fractions were separated by electrophoresis in a nondenaturing gel. Lanes 19 and 20 are negative controls without protein and with 10 μM GST tag, respectively. (c) Quantitative analysis of EMSA gels showing the effects of mutations on binding. Band shifts (% diminution of the free HJ bands) from three independent experiments were quantitated and are plotted against protein concentration, with standard deviations indicated.

To evaluate the BLM RQC-HJ binding model, we next performed binding assays using BLM RQCs mutagenized at putative HJ-binding residues. Four RQC variants were constructed, including the aforementioned loopΔ10 (a deletion mutant of the BLM-insertion) and alanine mutations of the putative DNA-binding residues S1121A, K1125A and R1139A. The four variants, in glutathione-S-transferase (GST)-fused form, were purified to homogeneity (Figure 7a) and then tested for binding by an electrophoretic mobility shift assay (EMSA).

The results are shown in Figure 7b and c. The wild-type protein bound to HJ in the micromolar range, while the loopΔ10 mutation resulted in a severe loss of binding (~50% loss at 5 μM). This loss is consistent with the present HJ-binding model in which the BLM-insertion interacts with a duplex arm of HJ (Figure 6a). On the other hand, the three alanine substitutions S1121A, K1125A and R1139A resulted in ~10%, 50% and 70% losses of binding, respectively, also indicating an involvement of the three wild-type residues in the RQC-HJ interaction. In particular, Arg1139, which is one of the P1-binding residues and probably forms a salt bridge with a backbone phosphate of HJ (Figure 5a), plays a crucial role in the interaction. This residue is conserved in BLM and RECQ1, but not in WRN (Figure 1b). In summary, all four of the BLM RQC variants resulted in a loss of HJ-binding ability, which supports the present binding model of BLM RQC to HJ.

## Discussion

The binding model of BLM RQC to a HJ (Figure 6a and b) offers the structural insights into the branch migration mechanism of BLM, in which DNA unwinding and annealing might be coordinated: a pair of acidic β-wings could catalyze strand separation in the east-west arms of HJ, whereas the basic BLM-insertions may help the unpaired strands to rapidly anneal with new partners by neutralizing negative charges on the north-south duplexes.

On the other hand, the recently determined NMR structure of BLM HRDC^7^,^8^ showed that the surface of the domain is extensively electronegative, implying a function for HRDC other than DNA binding. Indeed, purified BLM HRDC displays no detectable binding activity with either a forked duplex (Figure 4c; orange crosses) or HJ (Figure 7b and c). In Figure 6a and b, we connected the BLM HRDC structures to each C terminus of the two RQC domains with the 13-a.a. linker. Although the precise locations of the HRDC domains remain to be elucidated, the C-term extended loops in RQCs (encircled by dashed lines in Figure 6b) appear to guide the two HRDC domains into the two empty spaces between the horizontal and vertical arms of HJ.

In solution, HJ is known to adopt a dynamic structure between antiparallel stacked (Figure 6c) and open planar conformations (Figure 6d)^22^,^23^. The former is favored in the presence of divalent cations such as Mg^2+^, but is inhibitory to branch-migration reactions due to its closed structure, stabilized by strong van-der-Waals contacts and hydrogen bonds^24^,^25^. Therefore, proteins that promote branch migration must first open the four arms, as does RuvA, which binds exclusively to the open planar conformation of HJ^22^.

We propose that, in the case of BLM, the electronegative HRDC domain functions as a wedge to open the HJ arms by electrostatic repulsion. The C-term extended loop and the shortness of the 13-a.a. linker may both contribute to the spatial positioning of the HRDC domain in relation to HJ. Consistent with this concept, the BLM HRDC domain is required for the efficient dissolution of double HJs, but not for the unwinding of a normal forked duplex^20^.

The present study offers the first structural insights into the RQC domain of BLM. Novel structural motifs found in the domain, such as the acidic β-wing, the BLM-insertion, and the C-term extended loop, may contribute cooperatively to the specialized functions of BLM in relation to HJ. Importantly, neither WRN nor RECQ1, which both lack these motifs, can substitute for BLM in double HJ dissolution reactions^20^.

Interestingly, BLM RQC also binds to G-quadruplex (G4) DNA with high affinity^26^. The G4-unwinding activity of BLM, and also of WRN, may be important for the efficient replication of telomeres^2^,^27^,^28^,^29^. Future structural studies of BLM RQC should reveal the mechanisms by which the RQC domain acts on such a variety of abnormal DNA structures.

## Methods

### Protein expression and purification

DNA encoding BLM RQC (a.a. 1068–1209) was cloned from human BLM and inserted into the GST-fusion vector pGEX-6P-3 (GE Healthcare Life Sciences). The integrity of the coding region was confirmed by DNA sequencing. The plasmid was transformed into *Escherichia coli* strain BL21-CodonPlus RIL (Stratagene), which was grown in LB medium supplemented with 100 μg/ml ampicillin and 50 μg/ml chloramphenicol at 37°C to an OD_660_ of 0.6. Expression was induced by the addition of isopropyl-β-D-thiogalactoside (IPTG) to 250 μM. Following incubation for another 24 h at 20°C, cells were pelleted by centrifugation and washed with 0.9% (w/v) NaCl solution.

For purification, cells were resuspended in 2× PBS (phosphate buffered saline) supplemented with 2 mM dithiothreitol (DTT), and disrupted by sonication in an ice bath. The cell suspension was clarified by ultracentrifugation and the supernatant was loaded onto a Glutathione Sepharose 4B affinity column (GE Healthcare). The column was washed with 10 mM Bis-Tris-HCl (pH 7.0), 150 mM NaCl and 1 mM DTT, and the protein was then eluted using the same buffer supplemented with 15 mM glutathione. The column effluent containing GST-BLM RQC was digested overnight at 4°C with PreScission protease (GE Healthcare). The product was loaded onto a HiTrap SP cation-exchange column (GE Healthcare) and eluted using a 0–500 mM NaCl gradient in a buffer comprising 10 mM MES-NaOH (pH 6.8) and 1 mM DTT. The effluent fractions were pooled and concentrated by centrifugation using an Amicon Ultra 10,000 molecular weight cut-off filter. The protein was finally passed through a Superdex 75 gel filtration column (GE Healthcare) equilibrated with 10 mM Tris-HCl (pH 7.5), 100 mM NaCl and 2 mM DTT. Peak fractions were pooled and concentrated to ~30 mg/ml. SDS-PAGE of the sample gave one major band corresponding to ~15 kDa (Supplementary Fig. S1), and analysis by matrix-assisted laser desorption/ionization time-of-flight mass spectrometry (MALDI-TOF MS; Bruker Daltonics) confirmed that the protein had been successfully purified without degradation. Five vector-derived residues (Gly-Pro-Leu-Gly-Ser) were present at the N terminus. Ten milligrams of purified RQC was obtained per liter of culture. The sample was frozen in liquid nitrogen and stored at −80°C until use.

SeMet-labeled BLM RQC was expressed in M9 medium containing SeMet under conditions that inhibited the methionine biosynthesis pathway. The protein was purified by the same procedures as described above, except that the buffers were supplemented with a higher concentration of DTT (4 mM). MALDI-TOF MS analysis confirmed that all of the five methionine residues in RQC had been successfully substituted by SeMet.

### Crystallization

Crystallization screening was carried out by the vapor-diffusion method at 20°C using commercial screening kits (Hampton Research and QIAGEN); the protein was mixed in a 1:1 ratio with the reservoir solution. Optimized crystallization conditions for the native and SeMet-labeled BLM RQCs included 20–25% PEG 4000, 50 mM sodium phosphate, 150 mM sodium acetate, 100 mM Tris-HCl (pH 8.4) and 15% (v/v) glycerol. Crystals of the BLM RQC-arsenate ion complex were obtained by substituting the sodium phosphate in the crystallization buffer with the same concentration (50 mM) of sodium arsenate.

### X-ray data collection, phasing, and refinement

For X-ray data collection, the crystals were flash-cooled and maintained at 100 K in a nitrogen stream during exposure to the X-ray beam. Diffraction was collected on the beamlines at SPring-8, Japan, and then indexed and merged using HKL2000^30^. Detailed statistics of the structure determinations are summarized in Table 1. Molecular replacement using the RQC domains of WRN^6^,^15^, RECQ1^9^ or *E. coli*^31^ as a search model failed, probably due to the lack of homology and structural differences between BLM and other proteins (see Results).

The phases for the BLM RQC-phosphate ion complex were determined using the MAD method with the SeMet crystal. Selenium positions were determined using SHELX^32^. MAD phases were calculated using SHARP (http://www.globalphasing.com) and subsequently improved by density modification using DM^33^. The structural model was built using Coot^33^ and refined using CNS^34^. The stereochemistry of the final structure was assessed using MolProbity^35^, in which only two and no residues in Mol-A and Mol-B, respectively, were flagged as outliers. The coordinates of eight selenium atoms, which had been used for phasing, were consistent with the positions of methionine side-chains in the final structure. Structural superimpositions were performed using LSQMAN^36^ and figures were prepared using PyMOL (DeLano Scientific).

For the BLM RQC-arsenate ion complex, the X-ray data were collected at two different wavelengths, 1.0000 and 1.0439 Å, which were used for structural refinement and calculation of an anomalous difference Fourier map, respectively.

### Analytical ultracentrifugation

Ultracentrifugation experiments were performed at 20°C using an Optima XL-A analytical ultracentrifuge (Beckman Instruments) equipped with an An-60ti rotor. For sedimentation-velocity analysis, purified BLM RQC was dissolved in 10 mM Tris-HCl (pH 7.5) and 0.1 M NaCl at a sample concentration of ~70 μM. The sample and reference buffer were injected into each side of a double-sector centerpiece and then centrifuged at 40,000 rpm. Radial absorbance scans at a wavelength of 280 nm were measured every 10 min, and were subsequently analyzed using SEDFIT^37^. The partial specific volume of the protein at 20°C was calculated to be 0.740 ml/g based on the amino acid composition.

For sedimentation-equilibrium analysis, three protein concentrations of BLM RQC (OD_280_ of ~0.2, 0.4 and 1.2, which correspond to 34, 67 and 200 μM BLM RQC, respectively) in the same buffer were prepared. The samples and reference buffer were injected into a six-sector centerpiece and centrifuged at 29,000 rpm. Following equilibration for 24 h, the radial absorbance was scanned and then fitted to the calculated curve from the ideal single-species model.

### Preparation of mutant proteins

Mutagenesis of BLM RQC was performed using a QuickChange II Site-Directed Mutagenesis kit (Stratagene). PCR was performed using GST-BLM RQC as the template, and the fidelity of the PCR reactions was confirmed by DNA sequencing. The resultant proteins were expressed and purified in GST-fused form by the same procedures as those used for the wild-type. SDS-PAGE and MALDI-TOF MS confirmed that no degradation had occurred during purification. Protein concentrations were adjusted to that of the wild-type by measuring OD_280_, and were confirmed by Bradford assays (Bio-Rad) and SDS-PAGE.

### Fluorescence polarization assay

Fluorescein isothiocyanate (FITC)-labeled oligonucleotide (forked duplex) was prepared as previously described^6^,^7^,^17^. Purified BLM RQCs (wild-type and loopΔ10 mutant) in GST-free form were mixed with DNA in a solution containing 10 mM Tris-HCl (pH 7.5), 25 mM NaCl and 1 mM DTT. The final protein concentrations ranged from 0 to 5 μM, while the DNA concentration in all samples was 100 pM. Protein-DNA interaction was examined by fluorescence polarization using a full-range Beacon 2000 system (Invitrogen) with 490 and 535 nm excitation and emission wavelengths, respectively. Each measurement was performed at 25 °C, repeated five times, and then averaged.

### CD

BLM RQC wild-type and loopΔ10 mutant proteins in GST-free form were prepared at a concentration of 0.1 mg/ml in 10 mM Tris-HCl (pH 7.5) and 25 mM NaCl. The samples (400 μl each) were analyzed at 10°C using a JASCO 720 W spectrometer. The solvent spectrum was subtracted from those of the protein solutions. Measurements were repeated eight times and averaged.

### Construction of BLM-HJ docking model

A structural model for the BLM-HJ complex was constructed *in silico*. First, the structures of BLM RQC (present structure) and BLM HRDC (PDB ID: 2RRD)^7^ were connected to each other with a 13-a.a. linker of random conformation using Coot^33^. The BLM RQC domain was then superimposed onto the previous model of a WRN RQC-HJ complex^6^ using LSQMAN^36^. The obtained BLM-HJ model exhibited no significant steric hindrance between the protein and either DNA or the other protein molecule, and no stereochemical outliers in the linker region.

### EMSA

Proteins in GST-fused form were incubated with 0.2 μM of FITC-labeled DNA substrate at 4°C for 30 min in binding buffer comprising 10 mM Tris-HCl (pH 8.0), 2 mM DTT, 1 mM EDTA and 20% glycerol. The samples (10 μl each) were then loaded on nondenaturing 7.5% polyacrylamide gels and electrophoresed at 4°C in 1× TAE buffer (40 mM Tris-acetate [pH 8.0] and 1 mM EDTA) at a constant 13.6 V/cm for 110 min. Band shifts were directly visualized using a fluorimaging analyzer (Fujifilm LAS-4000) with a fluorescence filter (Y515-Di), with an exposure time of 10 s for each gel (no band intensity was saturated). The experiments were done three times, and the gels shown are representative. Quantitative analyses of the band shifts were performed using ImageGauge (Fujifilm).

## Additional information

**Accession numbers**: Atomic coordinates and structural factors for the BLM RQC domain bound to a phosphate ion and an arsenate ion have been deposited in the Protein Data Bank under ID codes 3WE2 and 3WE3, respectively.

## Supplementary Material

Supplementary Information Supplementary Figures

## Figures and Tables

**Table 1:** Crystallographic data for the BLM RQC domain

**X-ray Data**						
	Native-Phosphate^a^	Native-Arsenate^a^		SeMet-Phosphate^a^		
Space group	*P*4_3_2_1_2	*P*4_3_2_1_2		*P*4_3_2_1_2		
Cell parameters [Å]	*a* = *b* = 59.22	*a* = *b* = 59.72		*a* = *b* = 59.21		
	*c* = 210.15	*c* = 209.68		*c* = 208.9		
*V*_M_ [Å^3^/Da]/*V*_sol_ [%]	2.83/56.6	2.87/57.2		2.81/56.3		
SPring-8 beam line	BL44XU	BL41XU		BL44XU		
Resolution [Å]^b^	20 − 2.7 (2.8 − 2.7)	20 − 2.9 (3.0 − 2.9)		20 − 2.9 (3.0 − 2.9)		
			Peak	Peak	Inflection	Remote
Wavelength [Å]	0.9000	1.0000	1.0439	0.9788	0.9791	0.9948
No. reflections (unique)	10,768	8,985	8,041	8,838	8,845	8,689
Completeness [%]	98.2 (87.6)	99.0 (93.5)	97.4 (82.8)	99.1 (92.2)	98.4 (85.7)	96.5 (70.2)
Redundancy	12.7 (8.7)	12.9 (9.5)	12.3 (7.4)	11.8 (9.3)	11.5 (8.9)	11.4 (8.8)
Mean *I*/σ*_I_*	14.7 (4.2)	14.5 (5.0)	13.5 (3.5)	13.8 (4.4)	13.7 (3.8)	13.1 (3.6)
*R*_merge_ [%]	5.5 (35.9)	7.5 (38.4)	8.7 (44.1)	9.0 (42.6)	7.9 (46.3)	7.7 (49.2)

**Phasing (Se-Met MAD)**			
No. heavy atom sites	8		
Figure of merit	0.52/0.44 (acentric/centric)		

**Refinement**			
	Native-Phosphate	Native-Arsenate	
No. atoms			
Protein^c^	950/878 (121/112 a.a.)	950/867 (121/110 a.a.)	
Ligand	5 (Phosphate ion)	5 (Arsenate ion)	
Solvent	6	6	
*R*_work_/*R*_free_ [%]^d^	23.7/27.1	26.9/29.5	
Ramachandran plot [%]^c^			
Favored	84.0/95.4	84.0/95.3	
Allowed	14.3/4.6	13.5/4.7	
Outliers	1.7^e^/0	2.5^f^/0	
R.m.s. deviations			
Bond lengths [Å]	0.008	0.011	
Bond angles [°]	1.413	1.312	
Average B-factor [Å^2^]			
Protein^c^	92.7/85.4	89.9/82.3	
Ligand	116.4 (Phosphate ion)	133.2 (Arsenate ion)	
Solvent	81.8	79.5	

^a^One crystal was used for each data set. The total oscillation range was 180°.

^b^Numbers in parentheses refer to statistics for the outer resolution shell.

^c^Statistics for Mol-A/Mol-B.

^d^*R*_work_ = Σ | | *F*_obs_ | − | *F*_calc_ | | / Σ | *F*_obs_ |. *R*_free_ is the same as *R*_work_ except that a 5% subset of all reflections was held aside throughout the refinement.

^e^Gly1096(Mol-A) and Lys1176(A).

^f^Gly1120(A), Gly1129(A) and Lys1176(A).
