# Supplementary material for: Structure of the RecQ C-terminal Domain of Human Bloom Syndrome Protein
Source: Sci Rep. 2013 Nov 21;3:3294. doi: 10.1038/srep03294 (PMC6505963; doi:10.1038/srep03294)
Supplement: Supplementary Information — Supplementary Figures [file srep03294-s1.pdf]

Structure of the RecQ C-terminal Domain of Human Bloom Syndrome Protein.

Sun-Yong Kim, Toshio Hakoshima & Ken Kitano

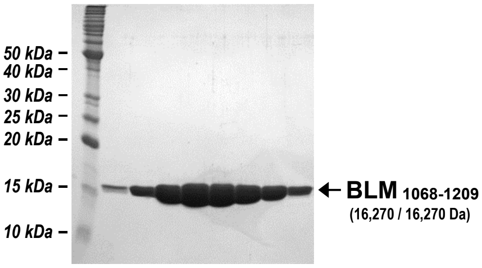

Supplementary Fig. S1.

SDS-PAGE of purified BLM RQC.

Fractions from gel-filtration chromatography, using a HiLoad 26/60 Superdex 75pg column (GE Healthcare), were analyzed by SDS-PAGE (15% polyacrylamide gel, stained with Coomassie Blue). The sample was also analyzed by TOF-MS to compare the mass with the calculated mass based on the primary sequence (TOF-MS / calc., in parentheses).

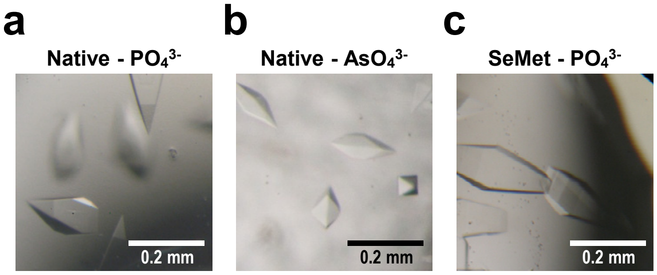

Supplementary Fig. S2.

Crystallization of BLM RQC.

(a) Crystals of BLM RQC in complex with the phosphate ion. **(b)** Crystals of BLM RQC in complex with the arsenate ion. **(c)** Crystals of SeMet-labeled BLM RQC in complex with the phosphate ion.

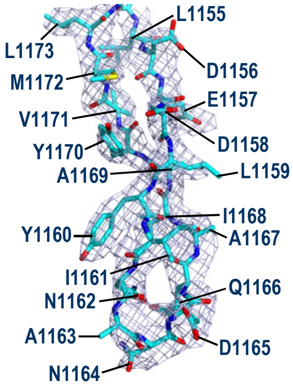

Supplementary Fig. S3.

Electron densities of the BLM β-wing.

A composite-omit map contoured at 1.0 σ is superimposed (Mol-A). The BLM β-wing, comprising 19 residues, is slightly longer than that of WRN (16 residues).

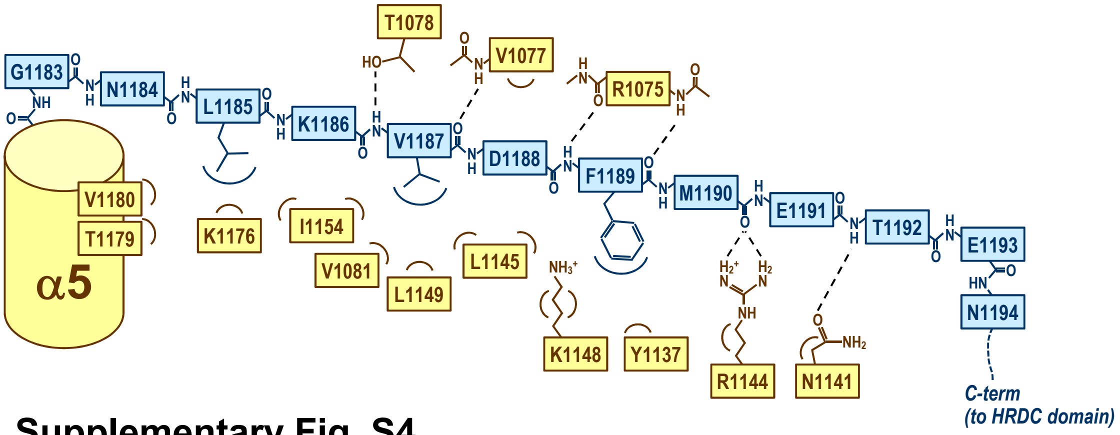

Supplementary Fig. S4.

Interactions between the C-term extended loop (blue) and RQC core (yellow).

Hydrophobic interactions are shown as curves and hydrogen bonds (< 3.3 Å) as dashed lines.
